# The flavors of plasma cells

**DOI:** 10.18632/oncotarget.4631

**Published:** 2015-06-28

**Authors:** Lynn M. Corcoran, Stephen L. Nutt

**Affiliations:** Molecular Immunology Division, The Walter and Eliza Hall Institute of Medical Research, and The Department of Medical Biology, University of Melbourne, Parkville, Victoria, Australia

**Keywords:** Immunology Section, Immunity, Immune response

As an object for study, the adaptive immune system is proving one of the most compelling and fascinating in the body. This system underlies our primary defence against acute infection and mediates long-term immunity by generation of memory and long-lived effector cells. It is notorious for its complexity and many diseases are associated with its deregulation, including autoimmune conditions, cancers, allergies and immunodeficiency. The clear health implications that insights into the inner workings of the immune system bring make this an important area of medical research.

To better understand this system, we adopted the strategy to first target one important cell subset for close examination as an exemplar for the whole system. Thus, we focussed our efforts on B cells, the generation of the plasma cell and regulation of antibody. The differentiation of naïve B cells into plasma cells is essential to producing the protective antibodies that enable us to fight infection. This developmental process also provides protection against re-exposure with the same infectious agent, through the production of long-lived memory B cells and plasma cells, and is the basis for virtually all currently used vaccination strategies. However, defects in plasma cell development can impact dramatically on human health, as evidenced by the plasma cell malignancy multiple myeloma (MM) [1] and antibody mediated autoimmune conditions such as Systemic lupus erythematous, vasculitis and Sjögrens syndrome [2, 3]. While antibody based therapies to deplete B cells, such as Rituximab (anti-CD20 monoclonal antibody (mAb)), are very effective against lymphoma and some autoimmune conditions, the plasma cell compartment, which fails to express CD20, is left intact upon treatment, highlighting the need for plasma cell specific therapies.

The B cell to plasma cell transition represents a dramatic molecular switch, from a transcriptional program controlling the B cell phenotype (sensing antigen, cytokines and T helper cells), to the plasma cell phenotype dedicated to antibody production, secretion and long-term survival [5]. Previous microarray studies have highlighted the repression of genes that accompanies differentiation, including genes for the antigen receptor and its signalling partners, for MHCII and antigen presentation, for cell cycle and other aspects of B cell character [4]. This transcriptional repression was largely accepted as a pragmatic adaptation to the plasma cell's main job: transcribing, translating and processing immunoglobulin chains for high-level antibody secretion.

**Figure 1:**
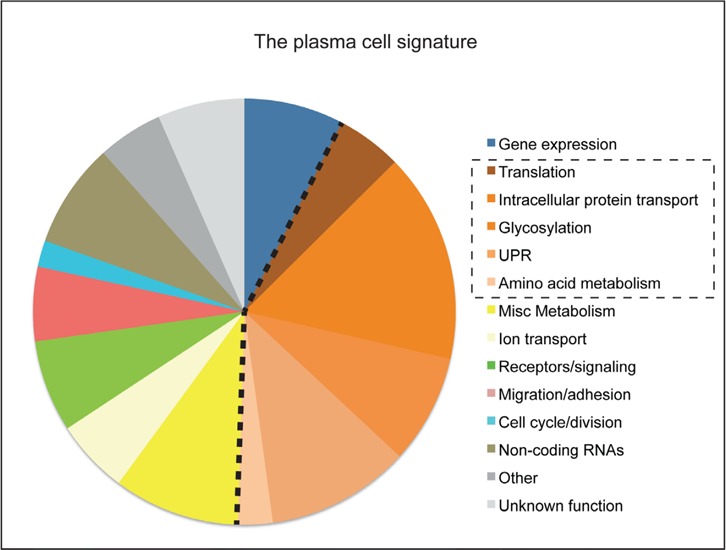
The functional annotations and proportional representation of genes in the 301 gene plasma cell signature are shown The are genes expressed at least 3-fold more highly in all plasma cells than peripheral B cells. Those that regulate protein synthesis, processing, trafficking and secretion are highlighted. They represent ~40% of the total signature. Immunoglobulin genes are excluded in this analysis.

We have recently performed a global analysis of the transcriptomes of most stages of late B cell and plasma cell differentiation from the mouse [6] and have used this data to derive a highly robust plasma cell transcriptional signature consisting of 301 genes (Figure 1). This signature includes the known markers and regulators of plasma cell biology, including *Sdc1/CD138*, *Blimp1/Prdm1* and *Xbp1,* as well as many previously identified transcripts whose function in the immune system has not been explored. Surprisingly, plasma cells actively transcribe as much of their genome as do naïve B cells. Nevertheless, in the most mature plasma cells of the bone marrow, around 75% of all mRNA encodes immunoglobulin. Interestingly, genes encoding several poorly known transcriptional regulators and signaling molecules are included in the signature. Several of these putative new players in plasma cell biology showed the same patterns of expression as known “master regulators” of plasma cell differentiation and function, Blimp1 and Irf4.

We published this work, and the underlying RNAseq data, as a Resource Article [6], as it contained many potential new insights into plasma cell biology and heterogeneity that might interest other investigators. These include differential gene expression between immature splenic plasmablasts and mature bone marrow plasma cells; differences between plasmablasts derived under T cell-independent and -dependent conditions; differences between plasmablasts generated *in vitro* and the long-lived mature cells found *in vivo*; gene expression changes that accompany B cell division during differentiation *in vitro*. The latter is of interest because we have shown previously that the probability of plasma cell differentiation from B cells *in vitro* is tightly linked to cell division number [7].

Reassuringly, the plasma cell signature also contained genes encoding proteins that are in pre-clinical and clinical development as plasma cell specific therapies for MM, for example *SLC3A2/CD98* and *Slamf7/CS1* (Elotuzumab). We hypothesize that the plasma cell signature, which we aim to now confirm in analogous studies with primary human plasma cells, contains genes encoding new plasma cell-specific proteins, that it will teach us more about the nature and survival strategies of normal plasma cells, and will identify new potential therapeutic targets on their pathogenic counterparts.
